# Distributed Dendritic Processing Facilitates Object Detection: A Computational Analysis on the Visual System of the Fly

**DOI:** 10.1371/journal.pone.0003092

**Published:** 2008-08-28

**Authors:** Patrick Hennig, Ralf Möller, Martin Egelhaaf

**Affiliations:** 1 Department of Neurobiology, Universität Bielefeld, Bielefeld, Germany; 2 Department of Computer Enigneering & Center of Excellence “Cognitive Interaction Technology”, Universität Bielefeld, Bielefeld, Germany; 3 Fakultät für Biologie, Department. of Neurobiology & Center of Excellence “Cognitive Interaction Technology”, Universität Bielefeld, Bielefeld, Germany; Indiana University, United States of America

## Abstract

**Background:**

Detecting objects is an important task when moving through a natural environment. Flies, for example, may land on salient objects or may avoid collisions with them. The neuronal ensemble of Figure Detection cells (FD-cells) in the visual system of the fly is likely to be involved in controlling these behaviours, as these cells are more sensitive to objects than to extended background structures. Until now the computations in the presynaptic neuronal network of FD-cells and, in particular, the functional significance of the experimentally established distributed dendritic processing of excitatory and inhibitory inputs is not understood.

**Methodology/Principal Findings:**

We use model simulations to analyse the neuronal computations responsible for the preference of FD-cells for small objects. We employed a new modelling approach which allowed us to account for the spatial spread of electrical signals in the dendrites while avoiding detailed compartmental modelling. The models are based on available physiological and anatomical data. Three models were tested each implementing an inhibitory neural circuit, but differing by the spatial arrangement of the inhibitory interaction. Parameter optimisation with an evolutionary algorithm revealed that only distributed dendritic processing satisfies the constraints arising from electrophysiological experiments. In contrast to a direct dendro-dendritic inhibition of the FD-cell (Direct Distributed Inhibition model), an inhibition of its presynaptic retinotopic elements (Indirect Distributed Inhibition model) requires smaller changes in input resistance in the inhibited neurons during visual stimulation.

**Conclusions/Significance:**

Distributed dendritic inhibition of retinotopic elements as implemented in our Indirect Distributed Inhibition model is the most plausible wiring scheme for the neuronal circuit of FD-cells. This microcircuit is computationally similar to lateral inhibition between the retinotopic elements. Hence, distributed inhibition might be an alternative explanation of perceptual phenomena currently explained by lateral inhibition networks.

## Introduction

Moving through an environment requires gathering information about the spatial properties of the surroundings. Collisions with obstacles have to be avoided and objects that may serve as landmarks for orientation need to be detected. Collision avoidance does not require detailed information about the object properties. Rather, it may be sufficient to know that there is an object no matter what it is.

In a wide range of species visual interneurons have been found which preferentially respond to small objects in their receptive field (see for instance: [1]–[5] cat, [6]–[8] monkey, [9]–[11] pigeon, [12] toad, [13], [14] locust, [15] hoverfly, [16], [17] hawkmoth, [18]–[20] dragonfly, [21], [22] blowfly). These cells differ in the size of their receptive fields and the preferred size of the objects. For instance, object sensitive cells in dragonflies or hoverflies respond most strongly to objects as small as 1–2 degrees. With increasing object size, the response vanishes almost completely [20], [23], [24]. Other cells like the so-called FD-cells of blowflies respond best to objects with a width in the range of 6–12 degrees and still may respond, although at a considerably lower level, during wide-field motion [21], [22], [25], [26], [27].

FD-cells are assumed to obtain their sensitivity for small objects through inhibition from another cell with a large receptive field. The assumption is based on laser-ablation experiments that revealed for at least one type of FD-cell, the FD1-cell, that its object preference disappears after eliminating an inhibitory wide-field neuron in its input circuitry [28]. Although the receptive field of the inhibitory neuron is larger than that of the FD-cell, inhibition from outside the receptive field borders of the FD-cell is not necessary for tuning FD-cells to objects. This is because the width of the excitatory visual field of an FD-cell is much larger than the optimum object size [21], [25]. Although the mechanisms underlying object sensitivity of the FD-cell have not yet been unravelled in detail, simple models have been proposed that can explain a preference for objects comparable to that of FD-cells. These models comprise an output neuron, the FD-cell that receives retinotopic input, as well as input from an inhibitory neuron. The synaptic transmission between retinotopic input elements and the FD-cell was assumed to be nonlinear [25], [29], [30].

After these models were put forward, the mechanisms underlying object sensitivity have been further constrained by new anatomical and electrophysiological data: (1) There is now good evidence for spatially distributed interactions in the input circuit or on the dendrite of the FD-cells [31], [32], (2) the responses of FD-cells were found to depend on object and background velocity in a very peculiar way, in addition to the already known preference for objects [26].

The above mentioned models were recently modified to allow a simulated fly to track a small moving target in a virtual environment [33]. Note that this modified model was tuned to target tracking rather than to account for the electrophysiologically determined responses of FD-cells. Moreover, it did not take into account the evidence for the spatially distributed interactions in the input circuit of the FD-cells.

Using model simulations we analyse three different wiring schemes with respect to their ability to comply with the two above mentioned experimentally established constraints. For all wiring schemes we assume the same receptive field for the inhibitory neuron and the FD-cell. To adjust the models to the constraints imposed by the electrophysiological data, we optimised the model parameters by means of an optimisation method.

The aim of the study is to unravel fundamental computational principles underlying object sensitivity of FD-cells and putting forward electrophysiologically checkable predictions, but not to mimic the detailed neuronal circuitry. Therefore, we chose a new paradigm which relies on only few free model parameters and allows us to model dendritic signal spread within a dendro-dendritic wiring scheme at a relatively abstract level by spatial lowpass convolution (compare with [34]). This enables us to avoid the many assumptions that are required for detailed compartmental modelling of nerve cells (e.g. [35]).

## Methods

### Constraints

The analysed models are constrained by the available experimental data on the wiring of the input circuitry of the FD-neuron and the responses of the FD-cell to different conditions of object and background motion. In the following we will focus on the FD1-cell, the member of the FD-cell ensemble which has been characterised most thoroughly. For the sake of simplicity we will use the term FD-cell in the modelling part of this study without explicit reference to a specific FD-cell.

### Constraints imposed by the structure of the circuitry

**Figure 1:**
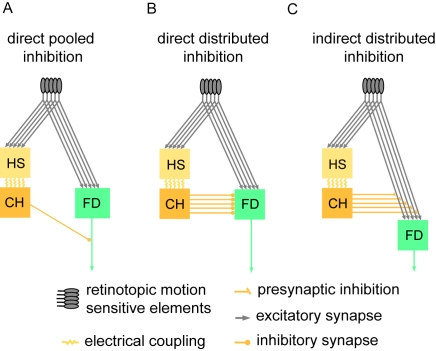
Schematics of potential circuits of the input organisation of an FD-cell. The small-field selective FD-cell receives excitatory retinotopic input from motion sensitive elements. Inhibitory input of the FD-cell is mediated by the vCH-cell via HS-cells. For simplicity, only one of the two HS-cells that provide input to the vCH-neuron is shown in this sketch. The coupling between the HS-cells and the vCH-cell is shown to be dendro-dendritic and occurs via gap junctions. A The vCH inhibits the FD-cell after spatial pooling (‘direct pooled inhibition’ DPI). B The vCH inhibits the FD-cell dendro-dendritically in a distributed way (‘direct distributed inhibition’, DDI). C The vCH inhibits the retinotopic input elements of the FD-cell in a distributed way (‘indirect distributed inhibition’, IDI).

The FD-cells are assumed to receive excitatory retinotopic input via their large dendritic trees from cells with small receptive fields encoding local motion information [21]. As assumed by Reichardt et al. [29] and Egelhaaf [25] and experimentally verified by Warzecha et al. [28], the FD1-cell is inhibited by a motion-sensitive cell with a large receptive field, the so-called ventral centrifugal horizontal cell (vCH-cell) (fig. 1). The interaction between the FD1-cell and the vCH-cell is likely to be spatially distributed (compare figs. 1A with 1B and 1C), because the vCH-cell's output area is large and has a profuse arborisation which largely overlaps the dendritic tree of the FD1-cell [32]. Until now it is not known whether the vCH-cell contacts the FD1-cell directly (fig. 1B) or whether the inhibition is presynaptic and thus indirect via the input elements of the FD1-cell (fig. 1C). The vCH-cell receives its ipsilateral excitatory input from dendro-dendritic electrical synapses from HS-cells (Horizontal System) [31]. The HS-cells are also motion-sensitive cells with a large receptive field and the same preferred direction as the FD1-cell but without a preference for small objects [36], [37]. Similar to the FD-cells, the HS-cells receive retinotopic input from local motion detectors. Hence, the ipsilateral inhibitory input of the FD1-cell is expected to be mediated via HS-cells and the vCH-cell.

### Characteristic response properties of FD-cells

The response of the FD1-cell to an object moving in front of a stationary background increases initially with an increasing object size. Beyond the optimum size of the object the response decreases again [25]. We will refer to this distinguishing property of FD-cells as “size dependence”.

Since both the FD1-cell and the inhibitory vCH-cell are motion- sensitive neurons, the velocities of object and background have a strong impact on the FD1-cell response [26]. For example, when the difference between the velocities of the background and the object decreases, the FD-cell response decreases. Moreover, a fast background and a slow object elicit stronger FD1-cell responses than an object with a moderate velocity in front of a stationary background. In the following, we will refer to the FD-cell's dependence on the object and background velocities as “velocity dependence”.

### Components of the model

#### Input organisation and receptive fields

As an input to the model FD-cell and the inhibiting element we used, as a first approximation, the one-dimensional velocity profile of the stimulus pattern along the horizontal extent of the visual field. For convenience, we did not explicitly model the properties of the retinotopic local movement detectors that are known to project onto the motion-sensitive tangential cells, such as FD-cells (review: [38]). The data of Kimmerle and Egelhaaf [26] suggest that the velocities used in their experiments were mostly restricted to the rising part of the velocity tuning curve. Hence the amplitude of the retinotopic input was assumed in our model simulations to be proportional to stimulus velocity. Since the objects used in the electrophysiological experiments which served as constraints for this study covered the entire vertical extent of the receptive field and were only moved horizontally, velocity differences were limited to the horizontal direction. Thus, taking only one spatial dimension into account does not represent a limitation. As we were mainly interested in finding a solution for the challenging problem of small-field tuning where the FD-cell and the inhibiting element have the same receptive field size, both elements were modelled with the same receptive field size which covered the entire pattern. For simplicity we neglected the experimentally determined spatial sensitivity distributions of the FD1- and vCH-cells, such that in our model both cells have the same sensitivity irrespective of the spatial location of the stimulus.

#### Distributed dendritic interaction as a lowpass filter

**Figure 2:**
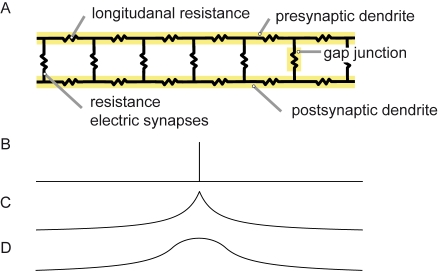
Dendro-dendritic blurring. A Simplified electrical equivalent circuit of dendro-dendritic coupling via electrical synapses. B An injected signal in the presynaptic dendrite C spreads electrotonically to the sides and gets spatially blurred. D The distributed coupling of both dendrites increases the blurring.

The distributed dendritic inhibition of the FD-cell's dendrites or its retinotopic input elements has been hypothesised to play an important role for the function of the FD-circuit [30]. If the dendrite is not only the input region of a neuron but also its output region, the activation pattern at the output reflects the input activation pattern to some extent. To get an intuition of the consequences of a dendritic arborisation for the retinotopic input activation pattern, one may imagine the dendrites of a neuron as an electric wire with a limited longitudinal conductance. A spatially localised input activity spreads to both sides along the dendrite (fig. 2). The signal amplitudes decrease with the distance from the input side and thus become spatially blurred [34]. This intuition may easily be generalised to two dimensions, if the fine dendritic branches show basically random orientations. The anatomy of vCH-cells appears to be not in contrast to this assumption [31], [39], [40]. Thus, the overall dendritic output of the vCH-cell can be described as a kind of spatially lowpass filtered version of its retinotopic input pattern. The spatial blurring of the retinotopic input pattern is further enhanced by the dendrodendritic interaction between the HS-cells und the vCH-cell.

Accordingly, we implemented the spatially distributed processing of the retinotopic input in the inhibitory part of the FD-cell circuit, consisting of HS-cells and the vCH-cell, as a single spatial lowpass filter. In the model these two cells are lumped into a single inhibitory element. In a first approximation, a rectangular filter kernel was used to spatially convolve the input signal. This approximation saves computation time since the filter can be calculated as a running average:
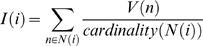
(1)with a neighbourhood *N*(*i*): = {*n*∶*i*−*σ*/2≤*n*≤*i*+*σ*/2; *1*≤*n*≤*W*}

V is the input signal and I the convolved output signal. i and n denote the position along the dendrite. W is the width of the receptive field and σ is the width of the filter kernel.

#### Spatial Integration

To unravel the significance of the spatially distributed processing in the neural circuit presynaptic to the FD-cell, the FD-cell is considered to be isopotential. The equivalent electrical circuit of a one-compartment passive membrane patch is used to calculate the membrane potential U_m_ of the FD-cell that results from spatial dendritic pooling:
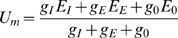
(2)E_I_ and E_E_ denote the reversal potentials of ion channels with the associated inhibitory (g_I_) and excitatory (g_E_) conductances, respectively. E_0_ is the resting potential of the cell. The inhibitory and excitatory conductances will be calculated from the respective input using functions specifying synaptic transmission between the presynaptic input and the corresponding postsynaptic cell (see below). The reversal potentials are fixed parameters. If the reversal potential of an ion channel is more positive than the resting potential E_0_ of the FD-cell, this channel is excitatory. A reversal potential more negative than the resting potential denotes an inhibitory channel.

We set the leak conductance g_0_ to 1. The other conductances are thus given relative to the leak conductance. The electrical equivalent circuit delivers a membrane potential U_m_ as a result.

#### Function of synaptic transmission

Synapses were often found to transform the presynaptic signal nonlinearly into postsynaptic responses [41]. Accordingly, we selected a sigmoid function which allows us to describe a broad range of characteristics by using only three parameters.
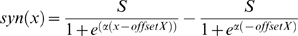
(3)The parameter α describes the slope, S accounts for the level of saturation and offsetX is used to specify which part of the function is taken as the operating range. The input argument x is always positive. For offsetX = 0 the function is approximately linear in the beginning. For offsetX>0 it approximates a saturation nonlinearity and for offsetX<0 initially a convex shape. The second part of the equation ensures that the function of synaptic transmission begins in the point of origin (syn(0) = 0).

### Direct Pooled Inhibition Model

**Figure 3:**
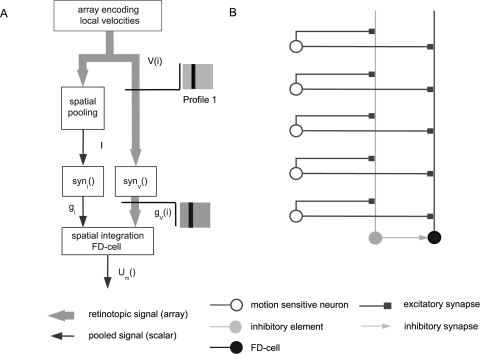
Direct pooled inhibition model (DPI). A Sketch of the DPI model: The motion picture (V(i)) provides the retinotopic visual input to the model with an amplitude at each position proportional to stimulus velocity. V(i) is the input for the (left) inhibitory and the (right) excitatory branch. Profile 1 illustrates the ‘motion picture’ which shows the spatial distribution of the velocity V(i) in the visual field of the object and the background (indicated by the grey level, the darker the higher the velocity). Hence, for each position i, a velocity value V(i) is given. In the left branch the values V(i) are spatially integrated to the signal I. This signal is transformed into the conductance g_I_ via the synaptic transmission function syn_I_(). In the right branch a conductance g_V_(i) is calculated for each position i from the motion picture using the synaptic transmission function syn_V_(). In the last step all conductances g_I_ and g_V_(i) are used to calculate the output of the model. B A detailed sketch of the DPI model circuit. Retinotopic motion sensitive cells excite the inhibitory element of the circuit as well as the FD-cell (black). The inhibitory element pools the retinotopic signal and inhibits the FD-cell directly. The symbols of the different cells are explained at the bottom of the figure.

The Direct Pooled Inhibition (DPI) model does not comply with the anatomical constraint of the inhibitory element conveying its signal in a distributed fashion to its postsynaptic targets. Nonetheless, this model will serve as a reference to understand the importance of a distributed processing. The FD-cell receives its excitatory and inhibitory input as a one-compartment passive membrane patch as described above. The FD-Cell is directly excited by the vector V(i) of retinotopically distributed velocity values (fig. 3B).

The stimulus velocity V(i) from each spatial position i is transformed, via the synaptic transmission function *syn_V_*, into a conductance g_V_(i) (fig. 3A). All local ion channel conductances with the reversal potential E_V_ are pooled according to equation 2 and account for the activation of the FD-cell. The reversal potential E_V_ is a free parameter of the model, but it is more positive than the resting potential E_0_.

The FD-cell receives its inhibitory input from a neuron which has the same receptive field as the FD-cell itself (fig. 3B). After complete spatial pooling of the motion information, the inhibitory element directly controls the conductance g_I_ of inhibitory FD-cell ion channels. As these channels are supposed to be inhibitory, their reversal potential has to be equal to or more negative than the resting potential. The case of a reversal potential equal to the resting potential represents so-called shunting inhibition. The reversal potentials are free model parameters. Hence, optimisation of the model will constrain the values of these potentials. g_I_ is calculated as the spatial average of V(i) transformed by the synaptic transmission function syn_I_() (equation 4). Therefore, all spatial information is lost in the inhibitory signal. Note that in the inhibitory pathway the synaptic transmission function is applied after spatial integration, whereas this function is applied to the excitatory input before integration.

In terms of spatial pooling this model is similar to a previous model of the FD-cells [25], [42].
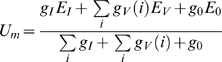
(4)with 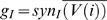 and *g_V_*(*i*) = *syn_V_*(*V*(*i*))

The DPI model has 8 free parameters: the reversal potentials E_V_, E_i_ and, for both functions of synaptic transmission, the three parameters characterising saturation, slope and position of the transmission characteristic (see above). For optimisation some parameters had to be constrained to ensure they are within a biologically realistic range. Thus, in the optimisation process, the reversal potential E_e_ is kept smaller than 100 mV whilst the reversal potential of the inhibitory ion channel E_i_ is held at the level of the resting potential (shunting inhibition) or at a more negative level (though not below −120 mV). It is obvious that the synaptically inducted conductances are biologically limited. Since we do not know the upper limit, we choose a wide range: The maximum of the excitatory and the inhibitory conductance are limited each to 10,000-times the leak conductance.

### Direct Distributed Inhibition Model

**Figure 4:**
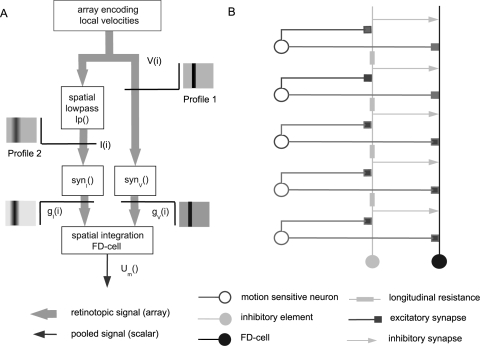
Direct distributed inhibition model (DDI). A Sketch of the DDI model: The motion picture (V(i)) provides the retinotopic visual input of the model with an amplitude at each position proportional to stimulus velocity. This motion picture (Profile 1) is the input of the (left) inhibitory and the (right) excitatory branch. For each position i, a velocity value V(i) is given. In the left branch the signal V(i) is spatially convolved with a rectangular lowpass filter kernel to lead to the signal I(i). Profile 2 illustrates the motion picture after spatial convolution. The signal I(i) is transformed by the synaptic transmission function syn_I_() into the conductance g_I_(i). For each position i a conductance g_V_(i) is calculated in the right branch. In the last step both the g_I_(i) and g_V_(i) conductances are used to calculate the output of the model. B Detailed sketch of the DDI model circuit. Retinotopic motion sensitive cells excite the inhibitory element of the circuit and the FD-cell. The inhibitory element inhibits the FD-cell directly in a spatially distributed way. The symbols of the different cells are explained at the bottom of the figure.

The Direct Distributed Inhibition (DDI) model uses the same activation of the FD-cell as the DPI model: the retinotopic velocity information V(i) is transformed into a sum of conductances g_V_ of excitatoy ion channels. However, DDI differs from DPI with respect to the inhibitory pathway (fig. 4A). DDI takes into account the evidence for a spatially distributed output of the inhibitory element. Furthermore, it assumes the inhibitory input of the FD-cell to be directly mediated via dendro-dendritic synapses between the inhibitory element and the FD-Cell (fig. 4B). Dendritic processing in the inhibitory element is modelled by a spatial lowpass filter as described above. The output of the inhibitory element, represented by the vector I(i), is retinotopically distributed. By applying the synaptic transmission function *syn_I_*, the output of the inhibitory element I(i) is transformed into an array of conductances g_I_(i) of the FD-cell (fig. 4A). In contrast to DPI, the retinotopic distribution of the inhibitory signal is preserved, though spatially blurred, until it reaches the FD-cell (compare figs. 3B and 4B).

The FD-cell integrates the velocity information in terms of the conductances g_V_(i) and g_I_(i), respectively. g_V_(i) and g_I_(i) are the conductances of the synaptically controlled ion channels with the reversal potentials E_V_ and E_I_, respectively:
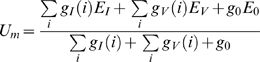
(5)with *g_I_*(*i*) = *syn_I_*(*I*(*i*)), *g_V_*(*i*) = *syn_V_*(*V*(*i*)) and I(i) as defined in eq. (1)

The model has 9 free parameters: the reversal potentials E_V_, E_I_, the width sigma of the filter kernel and, for both functions of synaptic transmission, the three parameters characterising saturation, the slope and the position of the transmission characteristic (see above). Here again the optimal values have been determined by optimisation (see below). The value ranges which are set to be valid for the optimisation are the same as for the DPI model.

From an abstract point of view, DPI is only a special version of DDI. If the inhibitory neuron of the FD-cell circuit were electrically compact, this neuron would have exactly the same potential along the entire dendrite. For the DDI model this situation is given for an infinite width of the spatial filter kernel averaging the signal across the entire receptive field.

### Indirect Distributed Inhibition Model

The Indirct Distributed inhibition (IDI) model differs from the other models in one essential aspect: the inhibition of the FD-Cell is indirect, since it is mediated via its presynaptic retinotopic input elements. Here, not the FD-cell itself is inhibited, but its input elements. As a first approximation, the inhibition is implemented as a pure shunting inhibition, i.e. E_i_ = E_0_.
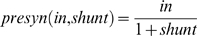
(6)
*in* is the input signal, *shunt* denotes the strength of the shunt.

**Figure 5:**
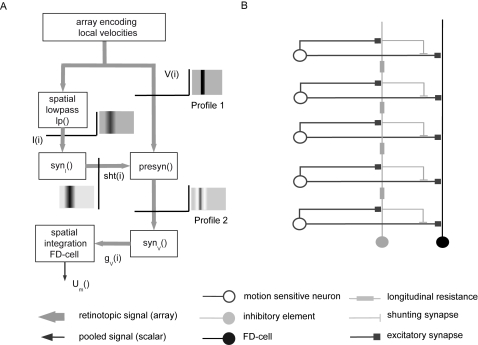
Indirect distributed inhibition model (IDI). A Sketch of the IDI model: The motion picture (V(i)) is the retinotopic visual input of the model having an amplitude proportional to stimulus velocity at each position. This motion picture (Profile 1) is the input of the (left) inhibitory and the (right) excitatory branch. For each position i, a velocity value V(i) is given. In the left branch the signal V(i) is spatially convolved with a rectangular lowpass filter kernel to lead to signal I(i). The signal I(i) is transformed by the synaptic transmission function syn_I_() into the shunting signal sht(i). For each position i, the signal V(i) is shunted by sht(i) and, by the synaptical transmission function syn_v_(), transformed into the conductances g_V_(i). Profile 2 illustrates the motion picture after the presynaptic inhibition. In the final step, all conductances g_V_(i) are used to calculate the output of the model. B Detailed sketch of the IDI model circuit. Retinotopic motion sensitive cells excite the inhibitory element of the circuit and the FD-cell. Before the motion sensitive elements reach the FD-cell, they are shunted in a spatially distributed way by the inhibitory element. The symbols of the different cells are explained at the bottom of the figure.

The inhibitory element contacts the output area of the local movement detectors and shunts them before they reach the FD-cell (fig. 5). The shunting is applied in a spatially distributed way. Again, in the inhibitory pathway we describe the effect of dendritic processing by spatial lowpass filtering of the array V(i) representing the retinotopic velocity values. Using the synaptic transmission function syn_I_() as specified above, the lowpass-filtered signal I(i) is transformed entry-wise into the shunting signal (see equation 7). This signal shunts the retinotopic input of the FD-cell according to Equation 6. Employing the synaptic transmission function syn_V_() the resulting signal is transformed into the array of local conductances g_v_(i) of the FD-Cell (fig. 5A). Similar to DDI, the FD-cell is implemented as a one-compartmental patch. The sum of g_V_(i) reflects the total conductance of the FD-cell corresponding to ion channels with the reversal potential E_V_. The FD-cell has no direct inhibitory input.
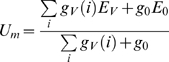
(7)with *g_V_*(*i*) = *syn_V_*(*presyn*(*V*(*i*), *syn_I_*(*I*(*i*)))) and I(i) as defined in eq. (1)

The model has 8 free parameters: the reversal potential E_V_, the width sigma of the spatial filter kernel and, for both functions of synaptic transmission, the three parameters characterising saturation, the slope and the position of the transmission characteristic. Again the optimal values have been determined by optimisation (see below). The value ranges which are set to be valid for the optimisation are the same as for the previous models.

### Optimisation

The model parameters were optimised to mimic the experimentally determined velocity and size dependences of the FD-cell response. Since only spike rates were available from the extracellular recordings of FD-cell activity, these had to be transformed into membrane potentials. Based on a previous study, the membrane potential was assumed to be proportional to the spike rate [43]. The proportional factor was estimated from sample intracellular and extracellular recordings [21]. Membrane potential depolarisations of about 20 mV were found when the cell fires at a rate of about 120 spikes per second. Further, a resting potential of −52 mV was estimated [21].

Our aim was to account for both the size dependence and the velocity dependence of the FD-cell response in each model using only a single set of parameters. Therefore, it was essential to optimise the models simultaneously with respect to both criteria. Since the experimentally determined dependence of the FD response on object and background velocity comprises more data points than the size dependence results (compare [25] with [26]), the former data would have a much greater impact on the optimisation result than the latter data. To compensate this effect we employed the following procedure: we modelled the size dependence of the FD response for two additional object velocities by using the experimentally determined size dependence and scaling the amplitude of the responses according to the velocity dependence experiments. In this way the characteristic size dependence of the FD-cell response had sufficient weight in the optimisation process. It should be noted that we did not try to obtain an exact fit of the experimental data, but only tried to account qualitatively for their characteristic features. Therefore, we used a distance measure which weights large deviations between biological and model data much more than small deviations. To calculate the overall distance between biological and model data the following distance measure d_rms_ root mean squared was chosen:
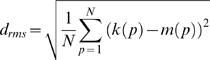
(8)A penalty term which was added to d_rms_ if a parameter is beyond the valid value range (see above) ensured the parameters to stay within this ranges.

To asses the significance of the smallest distance values obtained by the optimisation procedure we used a constant artificial response which has the smallest d_rms_ distance to the biological data as one reference. This reference assumes that the neuronal response does not depend at all on the tested stimulus parameters. The standard error of the mean (SEM) of the data from the velocity dependence experiments [26] was used as another reference.

### Algorithm

We applied “Differential Evolution” as an automatic stochastic optimisation method [44]. It is a convenient procedure for continuous, nonlinear and multimodal but analytically inaccessible functions. Systematic variations of the parameters did not reveal any discontinuities in the distance measure d_rms_ as a function of the model parameters. Consequently, we expect Differential Evolution to be an appropriate optimisation method.

The algorithm searches for the global optimum of the function to be analysed. In our case we want to find the optimum of the distance measure d_rms_ as a function of 8 (DPI and IDI models) or 9 (DDI model) parameters. Since Differential Evolution is a stochastic optimisation method, finding the global optimum is not guaranteed, as it is possible to get stuck in a local optimum. Initially, the optimisation was performed several times in preliminary tests with different initial parameters of the search algorithm. The set of these parameters performing best was chosen for the final optimisation (weighting factor F = 0.7; crossover constant CR = 0.9; number of parents NP = 100).

The optimisation procedure was repeated 1,000 times for each model. Each run was stopped after a fixed number of iterations (200,000) or if the improvement in terms of d_rms_ in the last 10,000 steps of searching was negligible (<0.01 mV). Each of the 1,000 runs delivers one set of model parameters as a solution which is a candidate for the global optimum.

Since the optimisation procedure may return only a local optimum as a solution, more than one optimum was found for each model. Each of the optima was found several times. Hence, the algorithm did not get stuck in a single local optimum and the different optima were found reliably. However, there is no guarantee that we found all local optima including the global optimum. Systematic variations of the model parameters around the best found solution ensured that the algorithm did not get stuck between optima as solutions actually turned out to be local optima.

In the following we evaluate the different solutions for each of the three models. At first the solution with the best d_rms_ is most interesting. However, since also qualitative properties are important, good solutions in terms of the distance measure d_rms_ may also be interesting, even if they are not the best.

## Results

### Direct Pooled Inhibition (DPI)

**Figure 6:**
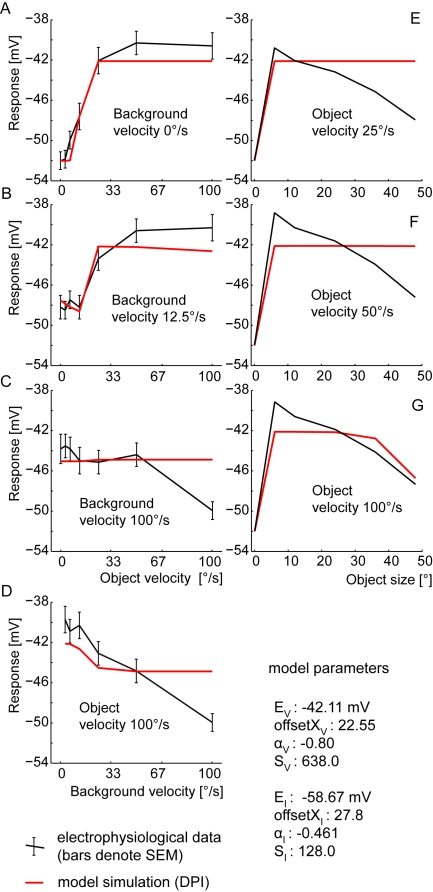
Performance of the DPI model. Best performance of DPI model with the model parameter S limited to 10,000. (The parameter S denotes a synaptically induced conductance relative to the leak conductance.) A–D Velocity dependence of the FD-cell response: model (red) and experimental (black) responses as a function of object velocity for three background velocities(a–c), respectively as a function of background velocity at a constant object velocity (d). Error bars denote the SEM of the electrophysiological data. E–G Size dependence of the FD-cell response: model (red) and experimental (black) responses as a function of object size for three different object velocities. (Experimental data taken from [25], [26].)

None of the solutions found for the DPI model mimics the size dependence of the FD responses: only for high object velocities the DPI responses decrease with an increasing object size. For small and medium object velocities, the model responses do not show any preference for small objects and the response is the same for all object sizes. On the other hand, all solutions for the DPI model mimic the velocity dependence quite well. The deviations are in the range of the SEM of the experimental data. Only at high background and object speeds do we find a big difference (fig. 6). The distance measure reflects the qualitative deviations: The best fit of the DPI model had a d_rms_ of 2.1 mV. This is beyond the SEM (1,2 mV) of the corresponding experimental data [26], but far below the d_rms_ of 3.7 mV for the constant response reference assuming that the response does not depend on the tested stimuli at all.

It was surprising that no pronounced small-field tuning has been obtained with DPI, since this distinguishing characteristic of FD-cells was previously obtained with a similar model [25]. When we optimised the DPI model solely with respect to the size dependence, we obtained also a clear preference for small objects. However, the model no longer mimics the dependence of the FD-cell on object and background velocity. These findings suggest that the DPI model, depending on the model parameters, can mimic either the characteristic size dependence or the object and background velocity dependence of the FD-cell, but not both characteristics simultaneously.

**Figure 7:**
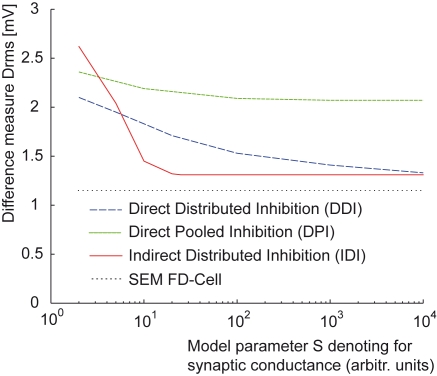
Consequences of increasing the inhibitory synaptic conductance. The distance measure d_rms_ for all models as a function of the model parameter S accounting for the maximum ratio between a synaptically induced conductance and the leak conductance. While DDI improves continually with an increasing synaptic conductance, IDI shows only clear improvements below a ratio of 10. For high ratios both DDI and IDI reach a distance measure near the SEM of the electrophysiological data (dotted line). DPI never gets close to the SEM of the experimental data [26].

Finding parameters leading to small d_rms_ is not sufficient for a model to be acceptable, it is also necessary that their optimised parameter values have biological plausibility. Three parameters of the best solution of the DPI model are at the border of the permitted range (see above). This is the case in one parameter vector for the parameter determining the slope of the synaptic transmission function and in another vector for the reversal potential of the inhibitory ion channels E_I_. Allowing values beyond this range did not noticeably improve the model. The third critical parameter is the level of saturation S of the synaptic transmission functions which is determined by the ratio between the synaptically induced conductance and the leak conductance of the FD-cell. With an increasing conductance ratio, the performance of the model increases slightly, but is no longer much affected for ratios above approximately 100 (fig. 7).

### Direct Distributed Inhibition (DDI)

**Figure 8:**
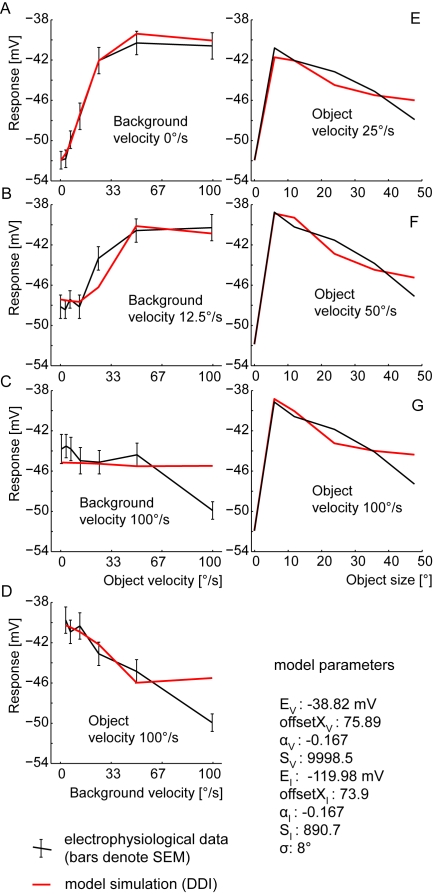
Performance of the DDI model. Best obtained performance of DDI model with the model parameter S limited to 10,000. (The parameter S denotes a synaptically induced conductance relative to the leak conductance.) A–D Velocity dependence of FD-cell response. E–G Size dependence of FD-cell response. Explanations as for Fig. 6.

For the DDI model we obtained three solutions which all proved to be better than those obtained with the DPI model. Each solution mimics the velocity dependence and the size dependence of the FD-cell responses quite well. Only for the data point at high background and object velocities do we find a large difference between experimental results and corresponding model response (fig. 8). However, small-field tuning is obtained for all velocities and the response of the model decreases with increasing object size for all velocities (fig. 8).

The obtained distance measure d_rms_ has values between 1.33 mV and 1.37 mV which are close to the experimentally determined velocity dependence SEM of 1.2 mV [26] (fig. 7). Hence, the deviations of the model from the experimental results are close to the range of variability of the experimental data.

Some parameters of the different solutions cover only a small range. The reversal potential of the excitatory input of all solutions is between −38.8 and −39.6 mV. Hence, the excitatory reversal potential is about 14 mV more positive than the resting potential. The inhibitory reversal potential is more negative than the resting potential. In different solutions it covers the large range between −56.4 and almost −120.0 mV, the border of the permitted parameter range. To test whether there is a significantly better solution beyond this border, we allowed the search to use parameters in wider confines. The solutions did not become significantly better. They improved by less than 0.01 mV in terms of d_rms_. In any case, the experimental data are explained best if inhibition does not represent a pure shunting inhibition, but has a pronounced subtractive effect. The width of the spatial filter reflecting dendritic blurring of the retinotopic signal was found for all solutions in the range between 8 and 12 degrees.

For all solutions the synaptic transmission functions have the shape of a sigmoid. We find almost the same shape of the synaptic transmission functions for the inhibitory and the excitatory synapses. The functions differ only in their saturation level and are just scaled by a factor.

The performance of the DDI model continually improves with an increasing conductance saturation level S up to the permitted limit of a ratio of 10,000∶1 between the synaptically induced conductances and the leak conductances (fig. 7). Decreasing parameter S below 100 the size dependence slowly vanishes. At an even smaller value of 10 the experimentally determined size and velocity dependences are not mimicked anymore.

### Indirect Distributed Inhibition (IDI)

**Figure 9:**
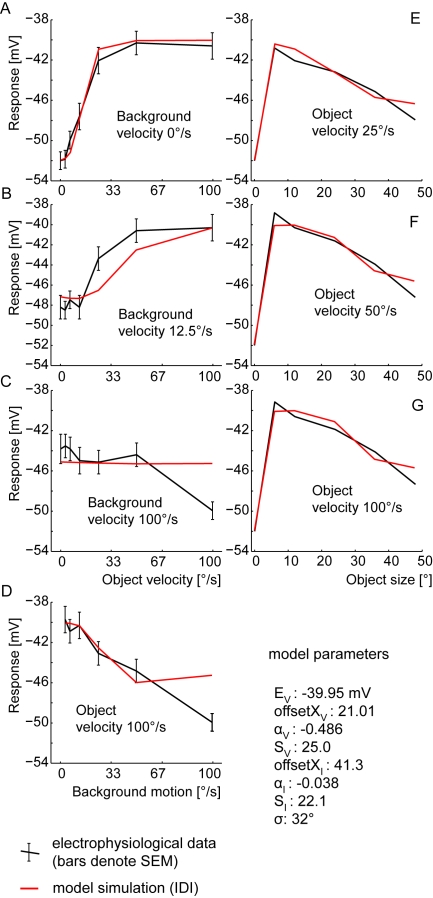
Performance of the IDI model. Best obtained performance of IDI model with the model parameter S limited to 25. (The parameter S denotes a synaptically induced conductance relative to the leak conductance.) A–D Velocity dependence of FD-cell response. E–G Size dependence of FD-cell response. Explanations as for Fig. 6.

Both the velocity and size dependence of the experimental data are fitted quite well by the model IDI, in a similar way to the DDI model. The four solutions found for the IDI model have, in terms of the distance measure, a performance similar to the DDI model (d_rms_ between 1.31 and 1.36 mV). Hence, the d_rms_ is close to the SEM of 1.2 mV as obtained for the corresponding experimental data. As for the DDI model, we observe a major deviation between the model and the experimental results only at the highest tested background and object velocities (fig. 9).

The reversal potential of the activating ion channels of the different solutions of the optimisation is in the range of −39.9 to −40.1 mV. This is close to the most positive membrane potential of the experimental data. Since we assumed a presynaptic shunting inhibition, there is no inhibitory reversal potential for the FD-cell itself. The width of the filter approximating the dendritic spread was between 32 to 34 degrees i.e. much broader than that of the DDI model.

For optimal performance of the model, the synaptic transmission function of the excitatory synapses was found to have the shape of a sigmoid, whereas the one of the inhibitory synapses is almost linear. As for the other two models, the performance of the IDI model improved with increasing parameter S accounting for the ratio between the synaptically induced conductance and the total leak conductance. In contrast to the other models, the performance improved quite strongly up to ratios as small as 10∶1 and improved only relatively little by further increasing the inhibitory conductance (fig. 7).

### Functional Principles

The optimisation procedure employed above reveals variants of the DDI and IDI models which account quite well for both the small-field tuning of FD-cells as well as for the dependence of their responses on the relative velocity of object and background. To get some insight into the functional principles relevant for the performance of these neural circuits, two aspects will be discussed with respect to DDI and IDI respectively.

#### Small field tuning based on DDI

**Figure 10:**
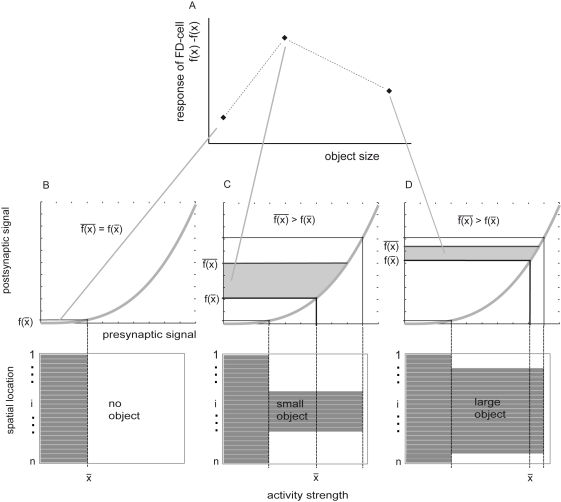
Preference for small objects with the same type of synaptic transmission function for the excitatory and inhibitory inputs of the FD-cell. A FD-cell response of the DDI model. In this model variant both the inhibitory and excitatory signal are transformed by an expansive synaptic transmission function. For both signals this function is the same (except for a constant scaling factor). The major difference is that the inhibitory signal is spatially integrated *before* applying the synaptic function, whereas the excitatory signal is integrated *afterwards*. The figure is to read from bottom to top. For illustration, imagine the integrating operation as an averaging of the input values x_i_ (in B,C,D top shown as spatial activity profile, i indicates the position) and the synaptic function as an expansive nonlinearity *f*() (grey curve, B,C,D top). Let the output be the difference between the excitatory signal 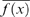 and the inhibitory signal *f*(*x̅*). For a homogeneous activity profile without an object (B bottom) the output is zero because 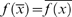 (B top). If some points peak out in the activity profile (C bottom), indicating a small object, the excitatory signal 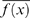 is bigger than the inhibitory signal *f*(*x̅*) (C top), the difference (grey area) is large. When the object covers almost the whole receptive field (D), the output (grey area) is larger than it is without an object, but is reduced compared with the smaller object. (A) Resulting response of the FD-cell as a function of object size.

In contrast to the early FD-cell model [25], [42], the solutions obtained for the DDI model with automatic parameter optimisation (see above) show that small-field tuning of FD-cells can be explained well by using similar expansive transmission functions for the excitatory and inhibitory synapses and without the assumption of a saturation of the inhibitory element or a shunting inhibition. The synaptic transmission functions of inhibitory and excitatory synapses differ only by a scaling factor. To get an intuitive idea of how a preference for small objects can be generated on the basis of the same type of synaptic transmission characteristic we have a closer look. The difference of the inhibitory and the excitatory input required for the preference for small objects arises from spatial blurring of the inhibitory retinotopic input signal and the exponential shape of the functions describing synaptic transmission, in particular, of the inhibitory synapse. With an increasing object size, the difference between the excitatory and the inhibitory signal profiles decreases (fig. 10).

Different characteristics of the functions of synaptic transmission of the inhibitory and the excitatory synapses are thus not essential and not a genuine functional principle of the circuit underlying a preference for small objects. It is only required that synaptic transmission operates according to an expansive nonlinearity, such as an exponential function.

#### Small field tuning based on IDI

The best solutions obtained with the IDI model (see above) are characterised by a nearly linear transmission of the inhibitory synapses, whereas the excitatory synapses have an expansive characteristic. Nevertheless, small field tuning may be obtained even with linear transmission characteristics at both excitatory and inhibitory synapses. This is illustrated here for a simplified model variant of IDI. It consists of a spatial low pass filter mimicking, as in IDI, the dendritic signal spread in the inhibitory neuron (see eq. 1), a shunting inhibition as given by eq. 6 and a linear summation accounting for both linear transmission of the input signals and the dendritic integration by the model FD-cell (model(input)):
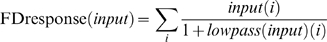
(9)


The entries of a spatially distributed signal input(i) account for the numerator of the fraction. The denominator consists of the entries of the spatially blurred input signal and a term accounting for the cells leak conductance. The leak parameter was arbitrarily set to 1.

**Figure 11:**
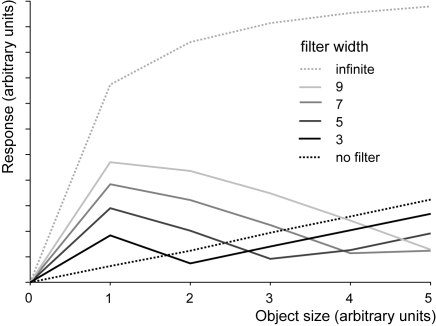
Consequences of the width of spatial dendritic blurring for small-field tuning of the FD-cell. Response of an FD-cell modelled according to IDI, but with linear synaptic transmission functions (see equation 9), as a function of object size. Parameter is the width of the filter mimicking the spatial blurring of the retinotopic input signal in the dendrite of the inhibitory element. With increasing width of the spatial filter, a preference for small objects emerges. The spatial width of the filter determines the optimal object size. If the filter width gets too large, the preference for small objects vanishes and for an infinite filter width the preference for small objects is completely lost (grey dashed line).

The model simulations reveal that just a spatial low-pass filter combined with a presynaptic shunting inhibition (and implicitly assumed linear transmission characteristics at all synapses) are sufficient to produce a preference for small objects (fig. 11). For numerical reasons this preference only shows up if some background activity of the input channels is assumed. This assumption is fairly plausible from a biological point of view.

Shunting inhibition of the retinotopic elements is by itself not sufficient to ensure small-field tuning of the model: Without spatial blurring the response of the inhibitory neuron the FD-cell response is proportional to object size (fig. 11). With increasing width of the spatial filter a preference of the FD-cell for small objects emerges. The spatial width of the filter determines the optimal object size. If the filter width gets too large, the preference for small objects vanishes and for an infinite filter width, i.e. for an isopotential inhibitory neuron, the preference for small objects is completely lost (fig. 11).

## Discussion

It has been the objective of this modelling study to challenge different model circuits with respect to their ability to account for a preference of FD-cells in the blowfly visual system for small moving objects as well as the characteristic dependence of their responses on object and background velocity [25], [26].

In all tested models small-field tuning is accomplished by inhibiting the FD-cell either directly or indirectly via another motion sensitive cell. Issues were the functional consequences of different architectures of the neuronal microcircuits. In particular we assessed the impact of localised inhibition after spatial pooling of retinotopic motion information versus distributed dendritic inhibition as well as pre- and postsynaptic synaptic interactions. We did this by employing a new approach of modelling the signal spread in a passive dendritic tree by spatial filtering of the cell's input activity pattern rather than by detailed compartmental models. The parameters characterising the three analysed model circuits were automatically optimised with respect to the most characteristic electrophysiological properties of FD-Cells. In contrast to inhibition after spatial pooling, circuits based on spatially distributed inhibition can approximate the preference of FD-cells for small objects and their dependence on object and background velocity so well that we are, in most cases, not able to clearly distinguish the experimental data from the model responses.

### The distributed inhibition satisfies all constraints

In the Direct Pooled Inhibition (DPI) model, the inhibitory element spatially integrates the motion signals before inhibiting the FD-cell directly. With appropriate parameter constellations it satisfies either the characteristic size dependence or the velocity dependence, but not both with the same parameter setting. Hence, this model cannot account for the characteristic features of the FD-cells.

With a spatially distributed interaction between the inhibitory element and the FD-cell or its input elements the performance improves significantly. In the model “Direct Distributed Inhibition” (DDI), the inhibitory element interacts with the FD-cell dendro-dendritically, whereas in the model “Indirect Distributed Inhibition” (IDI) it interacts presynaptically to the FD-cell with its retinotopic input elements. The two distributed models approximate quite well both the dependence of the FD responses on pattern size as well as its dependence on object and background velocity. At most data points are the model data within the standard error of the mean of the experimental data.

This good performance of the distributed models relies on a spatial blurring of the retinotopically mediated velocity signal in the dendrite of the inhibitory neuron. Hence, we can conclude that a distributed interaction which preserves the spatially distributed retinotopic velocity signal in the inhibitory neuron, though in a blurred form, is an essential part of the circuitry of object detection in the visual system of the fly.

This conclusion is in good accordance with the available experimental data: (1) elimination of the inhibitory vCH-cell eliminates the preference of the FD-cell for small objects [28]. (2) The vCH-cell and the FD-cell come in close contact to each other only in their dendritic regions. The dendritic aborisations of the FD-cell are totally covered by the aborisations of the vCH-cell along their horizontal extent [32]. (3) Varicose swellings on the dendrites of the vCH-cell indicate that the dendrites are an output region [40]. (4) A spatially distributed inhibition requires a distributed activation of the inhibiting neuron's aborisations when excited by spatially limited stimuli. This distributed activation was shown for the vCH-cell [32] and is likely to be mediated via dendrodendritic synapses by the so-called HS-cells [31]. (5) The joint input and output aborisations of the inhibitory vCH-Cell [32], [45], [46] form the structural basis of spatial blurring of the retinotopic input activity pattern [34].

### Advantages of distributed processing

As a potential advantage of a circuit relying on spatially distributed inhibition the inhibitory signal has more computational degrees of freedom than a pooled signal. In the latter case, only the signal strength can be varied as a function of time, whereas in the former situation the spatial domain can also be used. Hence, in the case of a distributed interaction the inhibitory signal may depend in different ways on object size as well as on the contrast and speed of the stimuli [30], [32].

There might be another advantage of distributed models (DDI and IDI) over a model where the signal in the inhibitory element is spatially pooled prior to its interaction with the FD-cell (DPI). Unlike the DPI model, when tuned to the size dependence of the FD-cell the responses of the DDI and IDI models do not confound two objects moving in the receptive field with a single object of twice the size in their responses. A second object has only a small effect on the response of the models with distributed inhibition, whereas the response of the DPI model decreases. This means that spatially distributed inhibition cannot be disturbed as easily as an inhibition after spatial pooling by a second object which turns up in the receptive field of the FD-cell. Although this prediction has not been tested in FD-cells so far, a similar effect was found in an object-sensitive cell of dragonflies [24].

### Indirect inhibition is less demanding

It is not known so far whether the spatially distributed inhibition operates directly on the dendrite of the FD-cell or indirectly via its retinotopic input elements. Both DDI and IDI are able to mimic similarly well all considered response properties of FD-cells. However, the synaptic conductance changes required for this performance differ for the two wiring schemes. IDI achieves the required performance with conductance changes which are by magnitudes smaller than the ones necessary for DDI. The performance of IDI does not improve further with increasing conductances. In contrast, DDI requires not only much higher conductance changes than IDI to satisfy the constraints, but gets continually better with growing conductance changes.

Measurements of input resistance in the axon of blowfly motion sensitive neurons without and during visual motion stimulation reveal a ratio of less than 2∶1 between the total synaptically induced conductance and the leak conductance [47], [48]. The FD-cell models proposed here hardly allow us to make realistic predictions of conductance ratios, because these models are intended to test the performance of different network architectures for a minimum set of assumptions and do not take the precise biophysical and geometrical properties of the involved neurons into account. Since in the electrophysiological experiment the postsynaptic sites are electrotonically distant from the recording site, the conductance changes determined in the axon may be considerably smaller than in the dendritic postsynaptic areas. This is because the conductance ratio in the axon depends on all conductances distributed over the dendritic tree, on the longitudinal conductances between the postsynaptic sites in the dendrite and the recording site in the axon as well as on the leak conductance of the axon (Hennig unpublished). Moreover, further geometrical properties may have to be taken into account: In the case of the DDI model, for example, the location of inhibitory synapses on the FD-cell's dendrite may affect the required conductances. An inhibition on the path between the retinotopic input sites and the axonal output site, was shown to be much more efficient than an inhibition in the more distal parts of the dendrites [49]. Thus, a closer analysis of the consequences of the spatial structure of the inhibitory neuron and the FD-cell requires detailed compartmental models with realistic biophysical parameters.

Nevertheless, independent of the biophysical details of the synaptic interaction between inhibitory neuron and FD-cell two advantages of a distributed indirect inhibition make this wiring scheme currently the most plausible one: Since IDI performs well for much smaller conductances than DDI, it is likely to be much less demanding with respect to energy expenditure. This is because, large synaptic currents require much more ions to be actively transported to the other side of the cell membrane. Furthermore IDI is less demanding with respect to the biophysical and geometrical properties of the FD-cell, because in DDI the very simple approximation of the model FD-cell operates sufficiently well only for very large synaptically controlled conductance. Only additional biophysical and geometric assumptions may – if at all - improve DDI in this respect.

### Prediction to distinguish indirect and direct inhibition electrophysiologically

The two distributed models might be directly distinguished by experimental analysis. Due to an indirect inhibition the overall conductance of the FD-cell should decrease with increasing object size. The overall conductance depends, apart from the leak conductance, on the excitatory synaptic conductances. Therefore, a decreasing cellular response with increasing object size is predicted to lead to a decreasing overall conductance. In the case of direct inhibition of the FD-cell however, motion in the receptive field would also lead to an opening of inhibitory ion channels in the FD-cell dendrite. With increasing object size, the inhibitory currents are predicted to overcompensate the excitatory input currents. Thus, in the case of a direct inhibition, the overall conductance of an FD-cell should increase with increasing object size, in contrast to an indirect inhibition.

### Open problems

Despite the good overall agreement of the models based on distributed inhibition and the experimental data, there are some differences for spatially extended objects and at high velocities.

The difference obtained for large objects may be caused by the very simplistic receptive field structure of the model cells. In the models we assumed the same sensitivity across the entire receptive field, although the sensitivity of real cells building the circuit declines towards the receptive field edges [21], [50]. Therefore, more realistic receptive field structures may improve some details of the model performance. Moreover, the receptive fields of both the model FD-cells and of the inhibitory element had the same size, whereas the inhibitory vCH-cells in real flies have a considerably larger receptive field extending even into the contralateral visual field [32], [50].

Two aspects may be responsible for the difference of the model performance and the experimental data at high velocities. (1) Velocity coding by biological motion detectors, as found in the fly visual system, is not linear [51]. The movement detector output first increases with increasing velocity, reaches an optimum and then decreases again. (2) The deviations between experimental and model results at high velocities may also result from the assumption of point symmetric synaptic functions. The synaptic functions may have been optimised to fit the data primarily at low velocities, since there are more data points corresponding to low velocities than data points at high velocities, resulting in a undesirable deviations at high velocities. These issues need to be tested on the basis of more elaborated model versions.

### Similarity to lateral inhibition

**Figure 12:**
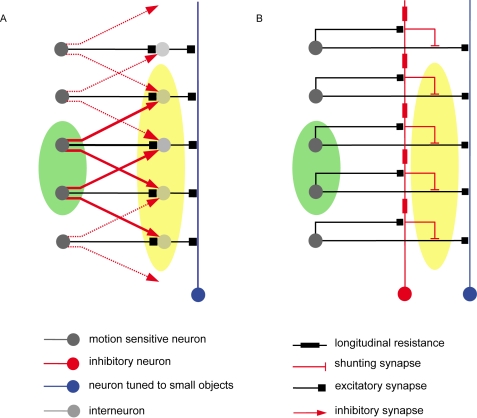
Lateral inhibition versus indirect distributed inhibition. A Lateral Inhibition circuit: retinotopic local interneurons (light grey) are directly inhibited by neighbouring local elements (dark grey). An excitation of the input elements indicated by the green area leads to an inhibition of the elements marked the yellow area. B IDI: inhibition is performed locally and indirectly via a neuron (red) with a spatially extended receptive field. As a consequence of dendritic blurring in the inhibitory element (red), the output cells are inhibited mainly within the neighbourhood of direct excitation. Here again an excitation of the input elements marked by the green area leads to an inhibition mainly in the area marked by yellow.

Spatial blurring of the retinotopic input resulting from dendritic signal spread in the inhibitory neuron is restricted to the neighbourhood of an activated input element. This is also true for the mechanism of lateral inhibition. A lateral inhibition circuit and the IDI model also show a structural similarity. In the case of lateral inhibition, a layer of interneurons is laterally inhibited by neighbouring input elements (fig. 12). Assuming appropriate parameter settings, the sum over the interneuron's activation shows a preference for small objects. In the IDI circuit, the layer of interneurons is replaced by a neuron with a dendritic output region that spatially blurs the retinotopic input signal. This signal then inhibits the input elements of the circuit's output neuron in a spatially distributed fashion. Hence, the mechanism of IDI is, to some extent, reminiscent of a lateral inhibition network. This functional similarity between the indirect distributed inhibition circuit and the lateral inhibition network suggests that sensory or perceptual phenomena that are conventionally be explained by lateral inhibition may be also accounted for in an alternative way. A classical example is the perceptual enhancement of contrast borders (often referred to as Mach bands [52], [53]). Whether a distributed dendritic interaction like the one presented with the IDI model is able to account in detail for this kind of phenomena needs to be tested.
